# Heterogenous oceanic redox conditions through the Ediacaran-Cambrian boundary limited the metazoan zonation

**DOI:** 10.1038/s41598-017-07904-3

**Published:** 2017-08-17

**Authors:** Junpeng Zhang, Tailiang Fan, Yuandong Zhang, Gary G. Lash, Yifan Li, Yue Wu

**Affiliations:** 1 CAS Key Laboratory of Economic Stratigraphy and Palaeogeography, Nanjing Institute of Geology and Palaeontology, Nanjing, 210008 China; 2 Nanjing Institute of Geology and Palaeontology, Chinese Academy of Sciences, Nanjing, 210008 China; 3 School of Energy Sources, China University of Geosciences, Beijing, 100083 China; 4 Department of Geology and Environmental Sciences, State University of New York, Fredonia, NY 14063 USA; 5 Sinopec Petroleum Exploration and Production Research Institute, Beijing, 100083 China

## Abstract

Recent studies have enhanced our understanding of the linkage of oxygenation and metazoan evolution in Early Cambrian time. However, little of this work has addressed the apparent lag of animal diversification and atmospheric oxygenation during this critical period of Earth history. This study utilizes the geochemical proxy and N isotope record of the Ediacaran–Cambrian boundary preserved in intra-shelf basin, slope, and slope basin deposits of the Yangtze Sea to assess the ocean redox state during the Early Cambrian metazoan radiation. Though ferruginous conditions appear to have prevailed through the water column during this time, episodes of local bottom-water anoxia extending into the photic-zone impacted the slope belt of the basin. Heterogenous oceanic redox conditions are expressed by trace element concentrations and Fe speciation, and spatial variation of N isotopes. We propose that the coupling of ocean chemistry and Early Cambrian animal diversification was not a simple cause-and-effect relationship, but rather a complex interaction. Specifically, it is likely that animal diversification expanded not only temporally but also spatially from the shallow shelf to deep-water environments in tandem with progressive oxygenation of the extensive continental margin.

## Introduction

The Cambrian explosion of diverse bilaterian clades is considered to have proceeded in three phases preserved in the fossil record: (1) the first phase is recognized by the appearance of basal metazoan phyla in Late Ediacaran time; (2) the second phase begins with the first occurrence of biomineralization of plates, spines and shells, and is recorded by the widespread occurrence of small shelly fossils (SSFs); (3) the final, perhaps the most profound, phase is defined by the emergence of the three supraphylogenetic clades during Cambrian stage 3^1^. Though these biological events have been attributed to environmental modifications, notably oxygenation of the global ocean, specifics of the relationship between the emergence and diversification of metazoans and atmospheric-oceanic oxygenation remain unresolved. Large-scale investigations have focused on the nature of geochemical constraints on atmospheric oxygen levels through geological time^2–7^ and (minimum) oxygen requirements for the stimulation and maintenance of metazoan evolution^8–11^. Conflicting conclusions of the necessity of oxygenation to the biological evolution of metazoans have been reported. Indeed, some have argued that increasing atmospheric oxygen levels might not have triggered the appearance of metazoans^12^. However, increasing concentrations of dissolved oxygen would have been required to sustain the subsequent diversification of metazoans and the establishment of a Phanerozoic-type ecosystem. Other studies have postulated that the activity of benthic faunas improved the chemical and physical conditions of their habitats by bioturbation and related bioirregation since the Fortunian stage^13^. Finally, although inferred atmospheric oxygen levels suggest that the global ocean may have been oxygenated by Late Ediacaran time, anoxic (perhaps intermittently euxinic) waters appear to have persisted in some areas of the Early Cambrian ocean.

A variety of geochemical approaches have been employed to characterize oceanic redox conditions during the Ediacaran–Cambrian boundary interval. Increasing δ^98/95^Mo from +1.7‰ at ~540 Ma to +2.3‰ (similar to modern seawater composition) by ~520 Ma suggests that bottom waters were fully oxygenated by Cambrian stage 3^14^. Fe speciation and S isotope data reflect the existence of a mostly ferruginous water column above a zone of limited bacterial sulfate reduction (BSR) in continental shelf and deep waters until Cambrian stage 3 in the Yangtze Sea^15–17^. Benthic redox conditions likely fluctuated frequently in intra-shelf basin areas of the Yangtze Sea as suggested by documented authigenic Mo-U co-variation^18^. Cerium anomalies documented from phosphate nodules recovered from basal Cambrian strata are consistent formation beneath an oxygenated water column^19^, yet the existence of photic-zone anoxia is suggested by largely negative sedimentary N isotope values described from Ediacaran-Cambrian deposits of the Yangtze platform^20, 21^. Thus, various geochemical proxies, some of them conflicting, suggest that redox history of Yangtze Sea was quite debatable at the time of the Cambrian explosion.

**Figure 1:**
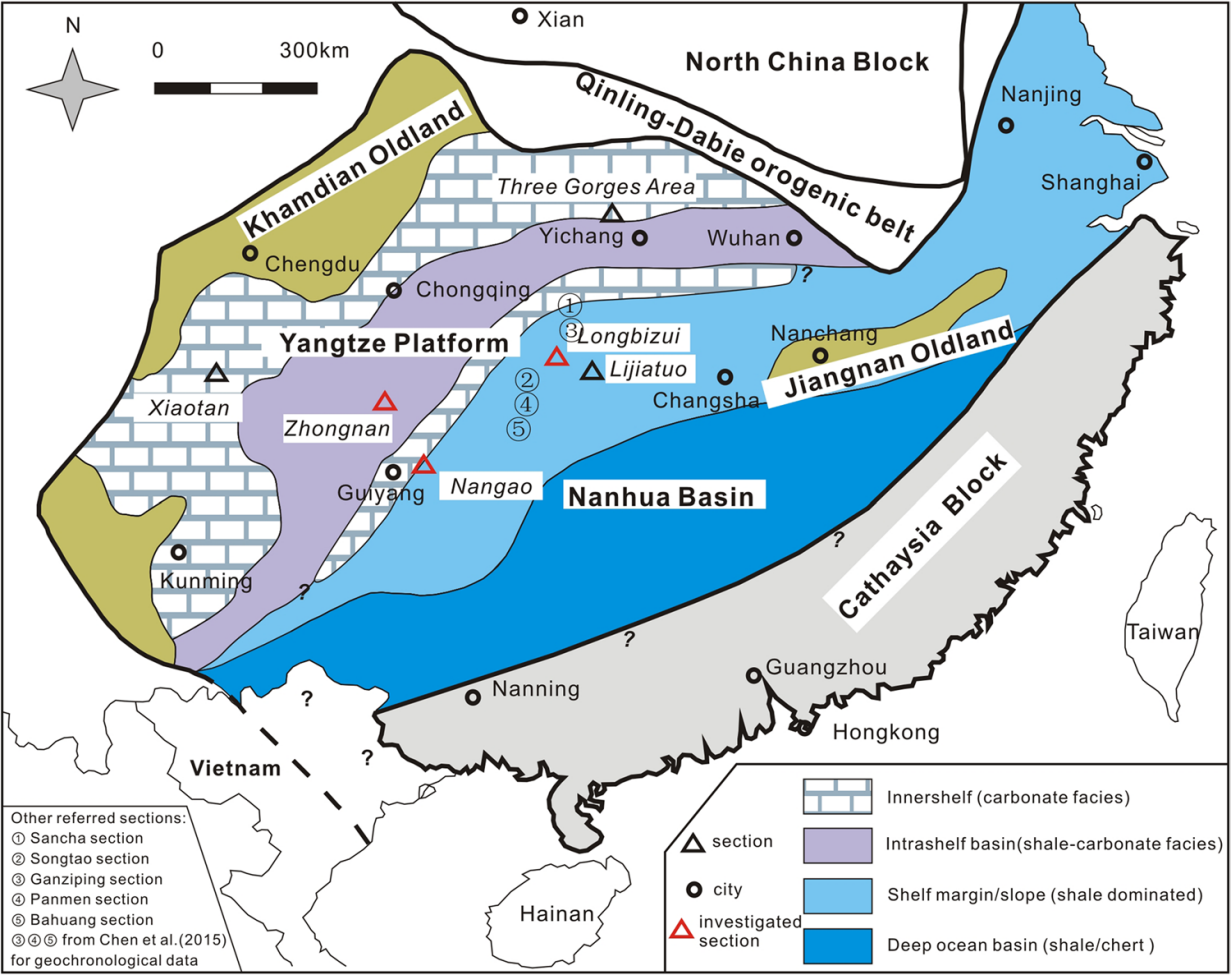
Paleoenvironmental map of the South China Block in the Early Cambrian (after ref. 18, with permission from Elsevier), showing locations of investigated sections. The figure was created by the author and co-authors, with mainly MapGIS 9.0 (http://www.mapgis.com/) constraining the geographic locations and CorelDRAW × 4 (http://www.corel.com/en/) further enhancing this illustration.

Herein, we address water column geochemistry of the Yangtze Sea during the Ediacaran-Cambrian transition using a combination of independent redox proxies, including N isotopes, trace element concentrations (mainly Mo and U), and Fe speciation. Application of this approach to the investigated sections, which were selected based on their inferred paleoceanographic positions within the Yangtze Sea (Fig. 1), elucidates spatial and temporal heterogeneity of ocean redox states. Longer-term geochemical trends are also addressed as a means of assessing the evolution of ocean chemistry through geologic time. A principal goal of the present study, then, is an improved understanding of the interaction of ocean redox state and the Cambrian metazoan explosion.

## Results

### Sedimentary N isotope records

**Figure 2:**
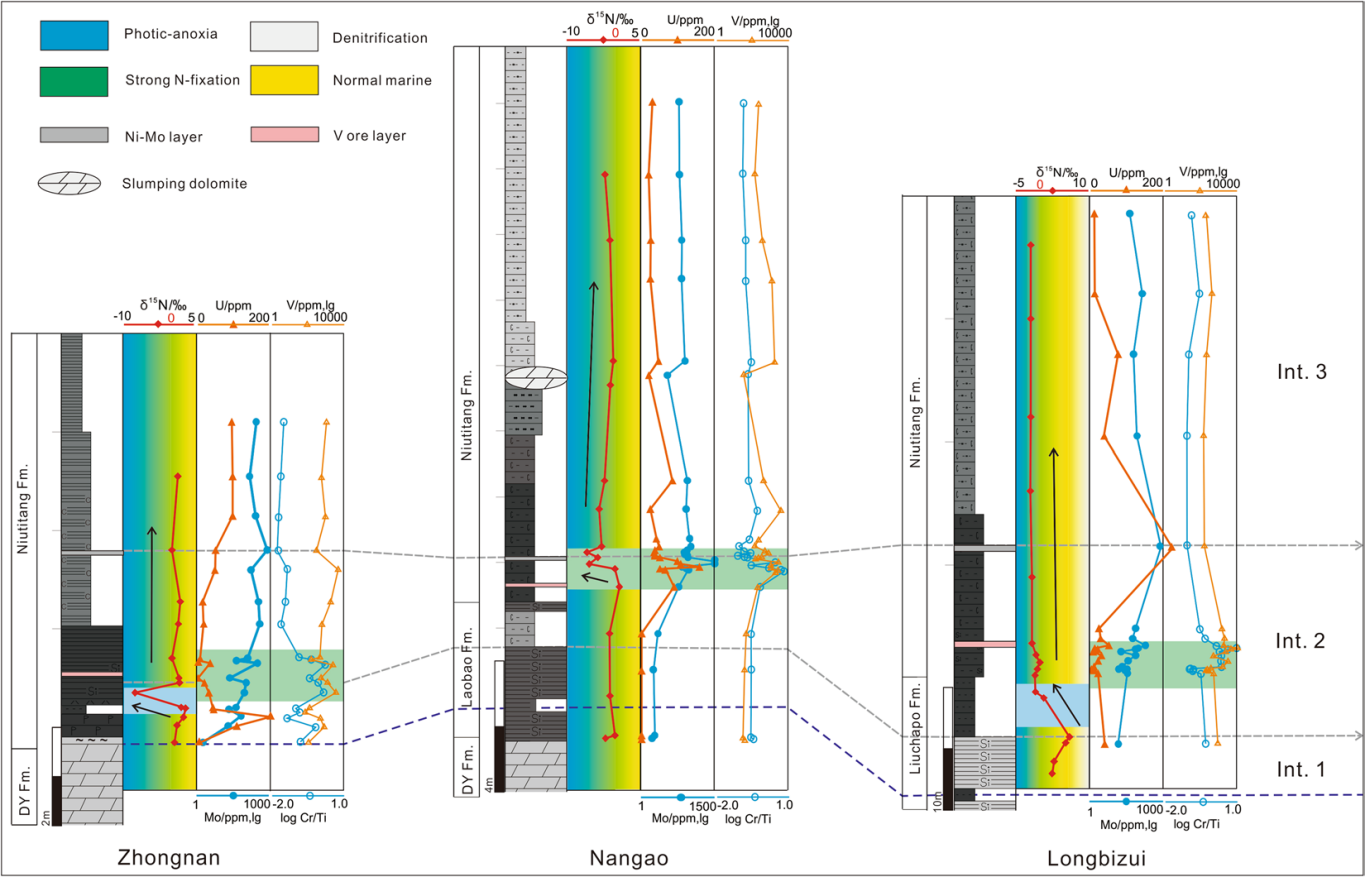
δ^15^N and redox-sensitive trace-metal concentrations for those investigated sections. Colored zones for N isotope profiles are used to show the transition from strong N-fixation to photic-zone anoxia. Three stratigraphic intervals are based on biostratigraphy and chemostratigraphy. See supplementary text for dividing details and Fig. S2 for lithology symbols. DY Fm. = Dengying Formation.

Reconstruction of the N cycle is essential to exploring the potential links between ocean chemistry and the marine ecosystem^22–25^. Sedimentary δ^15^N values of investigated samples range from −7.5‰ to +5.9‰, showing the greatest variation for all studied sections near the Ediacaran-Cambrian boundary (Fig. 2; refer to supplementary text for the stratigraphic and sedimentological context of the investigated intervals). The Zhongnan section displays consistently positive δ^15^N values with the exception of a single negative value obtained from a black shale sample (Fig. 2). The first interval in the Zhongnan section, which includes phosphate-rich deposits as well as intercalated chert and shale, is featured by values between +0.4‰ and 2.4‰, increasing slightly up-section (Fig. 2). Interval 2 is recognized by a sharp δ^15^N decrease of ~9.4‰, followed by a rapid return to positive values persisting into the third interval (Fig. 2). A subtle positive excursion appears at the onset of interval 3 that passes upward from a value of 0‰ near the Ni-Mo sulfide layer (Fig. 2).

Slope deposits of the Nangao section exhibit a δ^15^N profile different with that of the Zhongnan section. A weak positive excursion is observed near the bottom of interval 1 (Fig. 2). An overlying negative excursion in interval 2 is much smaller than its equivalent event in the Zhongnan section (Fig. 2). A much stronger negative excursion is documented just below the boundary of interval 2 and 3, immediately below the sulfide ore layer (Fig. 2). Above this, the δ^15^N profile displays a broad but subtle positive excursion of approximately 1.7‰ through interval 3 (Fig. 2). The δ^15^N profile of the Longbizui section displays generally lower values relative to the Zhongnan sections (Fig. 2). A positive excursion of ~5.9‰ occurs in the Liuchapo Formation of interval 1 (Fig. 2). A sharp negative shift by ~5.2‰ is observed at the beginning of interval 2. Further, negative δ^15^N values are concentrated in black shale in the interval 3 above the Ni-Mo sulfide layer^20, 21^.

Principal biogeochemical processes that influence N isotope composition include N-fixation by diazotrophic cyanobacteria, denitrification in the water column or within sediment and anaerobic ammonium oxidation (anammox)^24^. Assimilation of N_2_ into organic matter by N-fixation is accompanied by fractionation of as much as −4‰^26^. Denitrification of NO^3−^ to N_2_ is accompanied by^15^N-enrichment of the remaining NO^3−^ pool. Additional processes affecting N isotope composition include expansion of the oxygen-minimum zone and shoaling of the O_2_/H_2_S chemocline into the photic zone^27^. In each scenario, increased utilization of ^15^N-depleted ammonium by primary producers (dominantly eukaryotes) results in a reduction of δ^15^N values of accumulating sediment. Especially, marine sediments deposited during the OAEs may record striking ^15^N-depletion, with δ^15^N values commonly <−2‰ interpreted to be an expression of strongly reducing water conditions^28, 29^. Sharply negative δ^15^N deviations (by 2–4‰) from normal marine N cycling, then, can be considered as the evidence of photic-zone anoxia perhaps induced by a chemocline upward excursion^29^.

**Figure 3:**
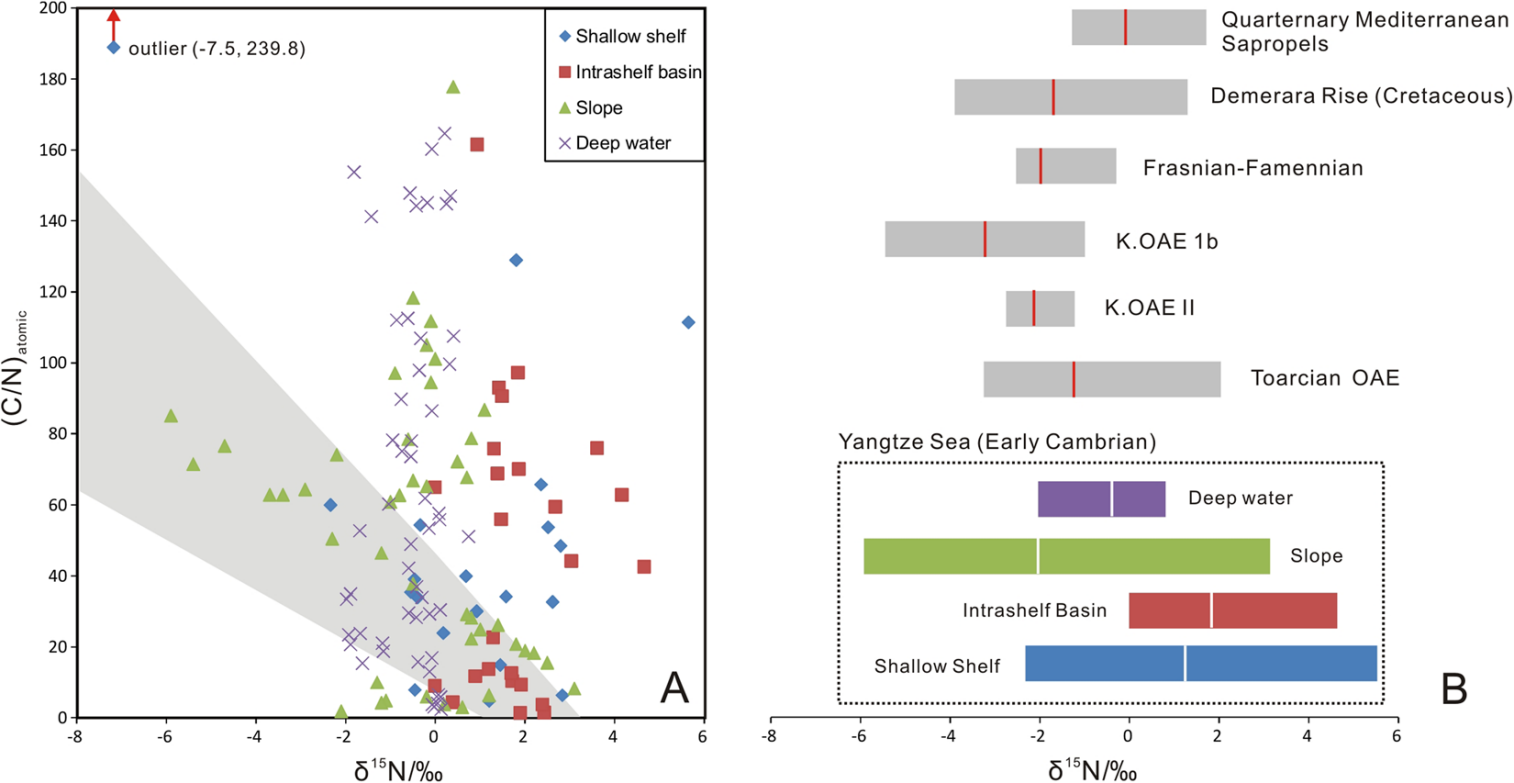
(**A**) Scatter plot of δ^15^N data and atomic C/N ratios from diverse depositional environments represented in various sections comprising Yangtze Sea deposits (refs 20, 21 and this study). The gray shade represents the range for those samples deposited during the OAEs^29^. (**B**) Comparisons of δ^15^N values recorded by marine sediments from the early-Cambrian Yangtze Sea and those from periods of widespread anoxia and OAEs (Data are sourced from refs 28 and 29). The gray box represents the range of values excluding outliers, with the line red/white in the box indicating mean values.

The N isotope composition of sedimentary organic matter can also be altered by diagenetic processes^24, 29^. The diagenetic loss of N from organic matter would be expected to yield C/N atomic ratios in excess of the 6.6 value of the Redfield ratio. Though the average C/N ratio of studied samples reaches 43.5, the weak correlation of δ^15^N and C/N (R^2^ = 0.0284) is not consistent with an interpretation involving strong diagenetic alteration of organic matter. Indeed, the weakly negative correlation (R^2^ = 0.3924) of δ^15^N and C/N values of the Nangao section suggests minor effect of diagenetic alteration on N isotopes (Fig. 3A). Further, given that the maximum isotopic deviation that can be ascribed to the selective removal of protein-N is generally no more than 2‰^29^, it is likely that the δ^15^N profile of the studied sections have been only minimally affected by diagenesis. It is noteworthy that N isotopes and C/N values described from modern and ancient shale successions, some of which accumulated during oceanic anoxia events (OAEs) are analogical to those of the present study (Fig. 3A). Thus the negative δ^15^N excursions documented from the sections spanning the Ediacaran-Cambrian boundary probably reflect some degree of modification of nitrogen cycling related to the shoaling of chemocline into the photic zone. The limited occurrences of negative δ15 N tend to argue against the establishment of photic zone euxinia. Instead, it is more likely that it was the expansion of anoxic bottom water into the photic zone that resulted in the consumption of ^15^N-depleted ammonium resulting in the negative δ^15^N excursions documented in sections along the slope belt, including the Nangao section. However, the possibility that photic zone euxinia occurred perhaps intermittently can’t be ruled out and should be addressed by further study of these deposits.

Details of N cycling preserved in a sedimentary deposit may reflect spatial variation of oceanic redox conditions from shallow to deep waters (Fig. 3B). Elevated δ^15^N values of approximately 0‰ recorded in the Zhongnan section may tell of episodes of N cycling in oxygenated surface water or slight denitrification within the water column below the oxic-suboxic boundary, as the stratified redox structure allows. However, the generally negative δ^15^N values common to slope deposits of the Nangao section could not have resulted from normal N cycling. Rather, such low values have originated from primary producers utilizing ^15^N-depleted NH_4_^+^, a process consistent with what is known of oceanic redox structure associated with the OAEs^26, 28^. The nearly consistent δ^15^N values of approximately −1‰ through interval 3 are suggestive of a redox-stratified ocean. As a redox-stratified ocean, the negative δ^15^N prevailed around −1‰ without significant excursions through the interval 3. Spatial variation of δ^15^N values appears inconsistent with widespread N cycling in late-Neoproterozoic ocean^25, 26^. Instead, anoxia may have been localized along the continental margin of Yangtze Sea, to some extent, lagging regional progressive oceanic oxygenation.

### Enrichments of redox-sensitive metals

Redox-sensitive metals, including Mo, U, V and Cr, are generally enriched under reducing bottom-water conditions^30^. The Zhongnan section exhibits variable, but enriched concentrations of these elements (Table S1 and Fig. 2). Mo concentrations range from 1.8 ppm in dolomite to 735 ppm associated with the sulfide layer. Mo is strongly enriched in the black shale, averaging approximately 300 ppm. Samples recovered from the basal Cambrian interval, including chert, are moderately enriched in Mo (19.2 ppm – 188 ppm). Uranium displays variable concentrations through the section from ~300 ppm in phosphate-rich deposits at the base of the Cambrian interval to ~30 ppm in overlying chert-bearing deposits, to 100 ppm in black shale higher in the succession (Fig. 2). V and Cr are strongly enriched in, and display generally sympathetic co-variance through, the Zhongnan section. However, V attains its highest value (3480 ppm) and Cr its lowest concentration (213 ppm) immediately below the Ni-Mo sulfide layer. Black shale samples of the Nangao section display lower enrichments of Mo (maximum = 149 ppm; average = 81 ppm) and U (maximum = 85 ppm; average = 37 ppm) than those of the Zhongnan section (Fig. 2). Those samples collected proximal to the Ni-Mo sulfide layer exhibit high concentrations of Mo and U (Fig. 2). It is noteworthy that the phosphate-enriched deposits of the Nangao section contain less U (<100 ppm) than equivalent rocks of the Zhongnan section (Fig. 2). The Longbizui section exhibits relatively low concentrations of Mo and U, yet higher contents of V and Cr. Chert samples of the Liuchapo Formation yield moderate concentrations of Mo (4–20 ppm) and low U (<3 ppm) (Fig. 2). Mo and U increase through the lower part of the Niutitang Formation of the Longbizui section, attaining maximum values (808 ppm and 331 pm, respectively) near the sulfide layer. V (maximum = 10,389 ppm) and Cr (maximum = 2,034 ppm) are strongly enriched through the interval of intercalated chert and shale of the Niutitang Formation.

**Figure 4:**
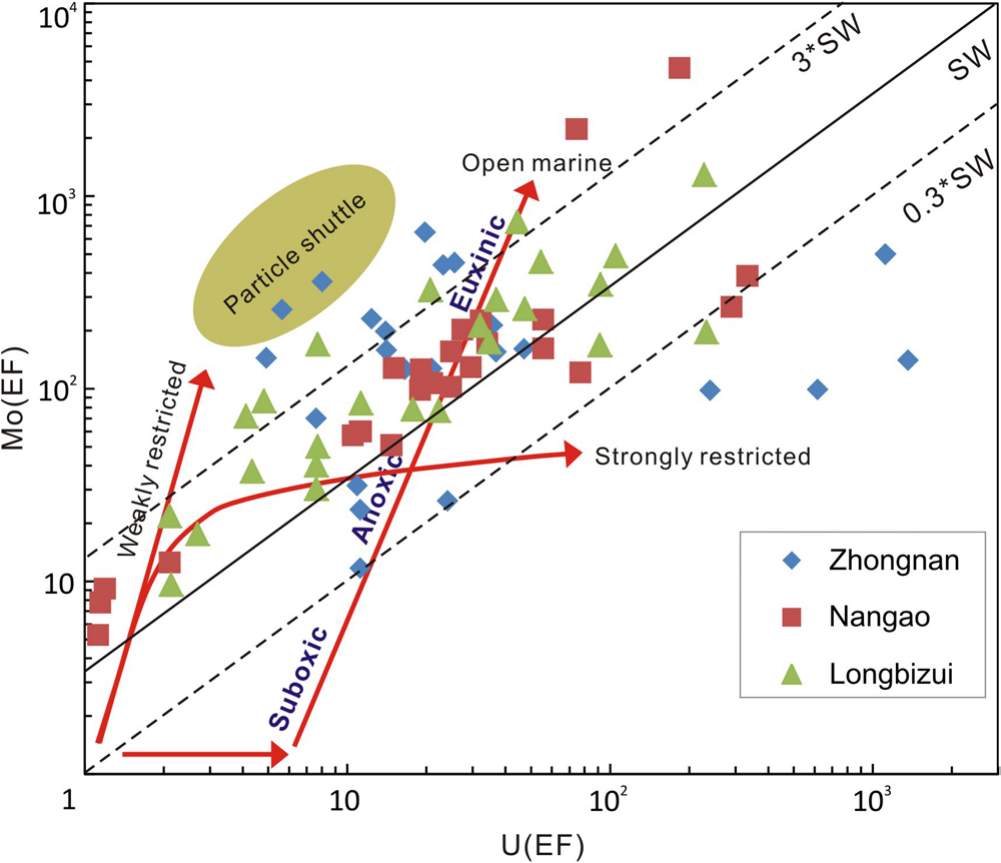
Plots to show Mo (EF) versus U (EF) for investigated sections. The solid diagonal line shows the seawater Mo/U molar ratio (1 × SW) and the dashed lines multiples thereof (3 × SW, 0.3 × SW). The olive-green field represents the “particulate shuttle” trend, characteristic of depositional systems in which intense redox cycling of metal, especially Mn oxyhydroxides occurs^33^.

Given that thiomolybdate formation requires free H_2_S^31, 32^, enhanced authigenic U uptake relative to Mo is to be expected under microbial-mediated conditions of higher redox potential, including suboxic or dysoxic conditions^33^. Organic-rich deposits of the studied sections display variable levels of U and Mo enrichment, suggesting anoxic to euxinic bottom water conditions (Fig. 4). Mo/U ratios of the Zhongnan section illustrate the greatest degree of scatter, varying from <0.3 × SW to >3 × SW, suggesting highly variable benthic redox conditions. Algeo and Tribovillard (2009)^33^ have interpreted Mo_EF_ and U_EF_ trends in terms of several modern ocean systems. According to this classification, the intra-shelf basin within which deposits of the Zhongnan section accumulated was likely weakly restricted^18^. Weak basin restriction may have led to stratification of the water column and establishment of anoxic or euxinic bottom-water conditions as suggested by Mo and U enrichments and negative δ^15^N excursions. This interpretation is consistent with previous studies that document negative ^98/95^Mo excursions documented from deposits immediately above the Ediacaran-Cambrian boundary tell of a restricted water column depleted of Mo^34, 35^. Shale deposits that accumulated in deep-water basin, the shale yield also high enrichments of Mo, U and redox-sensitive metals, indicating anoxic to euxinic bottom-water conditions even without hard evidences recording the existence of H_2_S in the water column. But the Mo-U covariation trends for deep-water sections display narrower slope ranges than that of Zhongnan sections, suggesting relatively stable bottom-water redox conditions (Fig. 4).

Enrichments of other redox-sensitive metals, including V and Cr, vary synchronously with Mo and U. Most Cr/Ti ratios of the investigated sediments are much greater than the crustal value of 0.0202 (calculated from modern crustal concentrations), suggesting that Cr enrichment principally by scavenging of particle reactive Cr (III) phases under strongly reducing conditions. Relatively low Cr/Ti ratios documented from the upper Niutitang Formation (interval 3) reflects less reducing conditions. Accelerated uptake of redox-sensitive metals and the coeval development of strong negative excursions in each of the investigated sections suggest the establishment of widespread reducing conditions. However, anoxic conditions appear to have persisted in slope regions into interval 3 as recorded by elevated Cr/Ti ratios and enrichment of Mo, U and V (Fig. 2). While the V concentrations express interestingly similar stratigraphic trends with Cr/Ti ratios, providing more evidences to shed light on the redox history.

The documented temporal association of stable N isotopic excursions and periods of redox-sensitive trace metal enrichment is consistent with what is known of the oceanic redox structure, but its timing may vary across the basin. Interestingly, the onset of negative δ^15^N excursions in Zhongnan and Longbizui sections appear to have occurred somewhat earlier than strong enrichment of redox-sensitive metals (especially Mo) associated with intervals 1 and 2. The Fe proxy indicates that the global ocean was dominated by ferrugenous water through the late Ediacaran to interval 1^36^. Accelerated upwelling at this time enhanced surface water productivity^18^ thereby increasing the flux of organic matter to the ocean floor, which further reduced bottom-water conditions. The shoaling of anoxic water mass into the photic zone caused primary producers to consume^15^N-depleted NH_4_^+^ resulting in the distinctly negative δ^15^N values documented from the studied deposits. It appears that enrichment of redox-sensitive trace metals lagged behind the isotopic response, gradually increasing as euxinic bottom water conditions were established. As modern oceans indicate, the residence times of those geochemical indices are ~3 kyr for N isotope, ~780 kyr for Mo and ~480 kyr for U, respectively. Thus a reason for the lag is proposed that isotopic evidences respond to oceanic changes quicker than trace metals. Probably, global ocean began to yield the oceanic anoxia earlier than the local ocean that experienced reducing conditions and consequent accumulation of redox-sensitive metals. However, this lag is not recognized in the slope belt, including the Nangao section. It is plausible that a rapidly rising sea level caused anoxic bottom water to shoal into the photic-zone producing the negative isotope excursion. Enrichment of trace metals appears to have accelerated across the basin at this time^18, 34^. Following this event and the related negative δ^15^N excursion, N cycling in the intra-shelf basin (Zhongnan section) returned to a more normal state with the δ^15^N values varying between 0‰ and 2‰. However, euxinic water persisted in the slope and deep-water basin, with unscheduled shoaling, as indicated by negative δ^15^N values (mostly <−1‰) and high concentrations of Mo in sediments. Despite the branching in time probably resulted, both the N cycling and trace-metal enrichment respond to the fluctuating oceanic redox state and reflect evidently in their sedimentary evidences.

## Discussion

**Figure 5:**
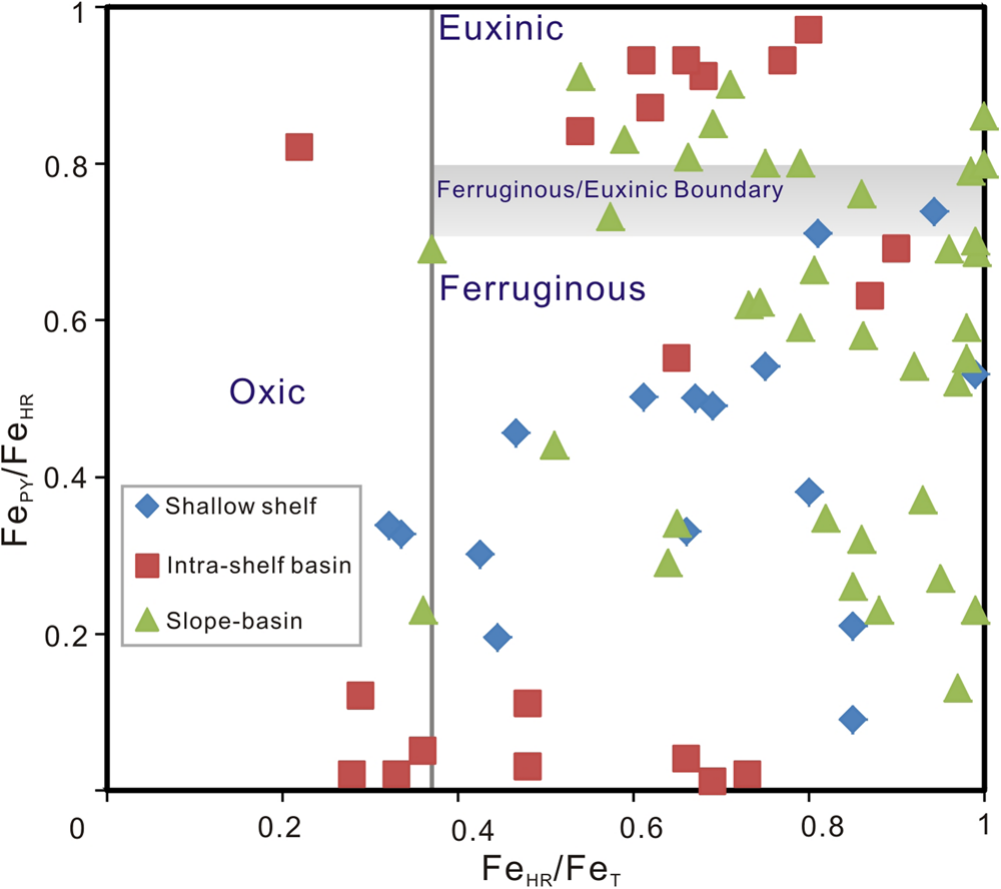
Fe speciation diagram for Cambrian samples from various depositional environments. Data are compiled from refs 14–16, 35 and 41.

Results of the present study suggest that areas of the Yangtze Sea experienced rapid fluctuations of oceanic redox conditions following the Cambrian explosion that may reflect lower than expected oceanic and atmospheric oxygen levels at this time. The Ediacaran biota may tell of increased atmospheric oxygen content and related widespread oceanic oxygenation following the Gaskiers glaciation (580 Ma). However, these conditions may have persisted for only several million years before the ocean, except for the mixed surface layer, returned to anoxic and ferruginous conditions that persisted well into the Early Cambrian^36^. During this time, though, the water column of the shallow shelf remained oxygenated, accumulating widespread carbonate sediment as exemplified by the Xiaotan section in the eastern Yunnan province and studied sections in the Three Gorge Area (Fig. 1)^20, 37^. Intra-shelf basins, including the depositional site of the Zhongnan section, became anoxic or even euxinic, as suggested by the present and previous investigations^18, 35^. Further, the presence of largely negative δ^15^N excursions as well as elevated Mo/U and Fe_py_/Fe_HR_ ratios observed in the studied sections may reflect local establishment of photic zone anoxia. Water column conditions of the slope area of the basin seaward represented by the Nangao section appear to have remained strongly reducing when the water column got improved elsewhere. Clearly, δ^15^N profiles of the deeper water Nangao and Longbizui sections display more consistently negative values than are documented from the intra-shelf Zhongnan section (Fig. 2) suggesting that these deposits experienced a protracted period of strongly reducing conditions. It should be noted that, however, iron speciation data from previous studies suggest that bottom–water conditions of the slope-to-basin region of the basin was seldom euxinic (Fig. 5). In summary, the commonly ferrugenous sub-photic water column of the Yangtze Sea margin experienced local anoxia or euxinia in intra-shelf basin and upper slope regions of the basin. The postulated redox state of the Yangtze Sea may have persisted until the ocean was fully oxygenated^17, 37^. Such diverse paleo-marine environmental conditions characterized the continental shelf of the Yangtze Sea in the face of the Cambrian explosion.

**Figure 6:**
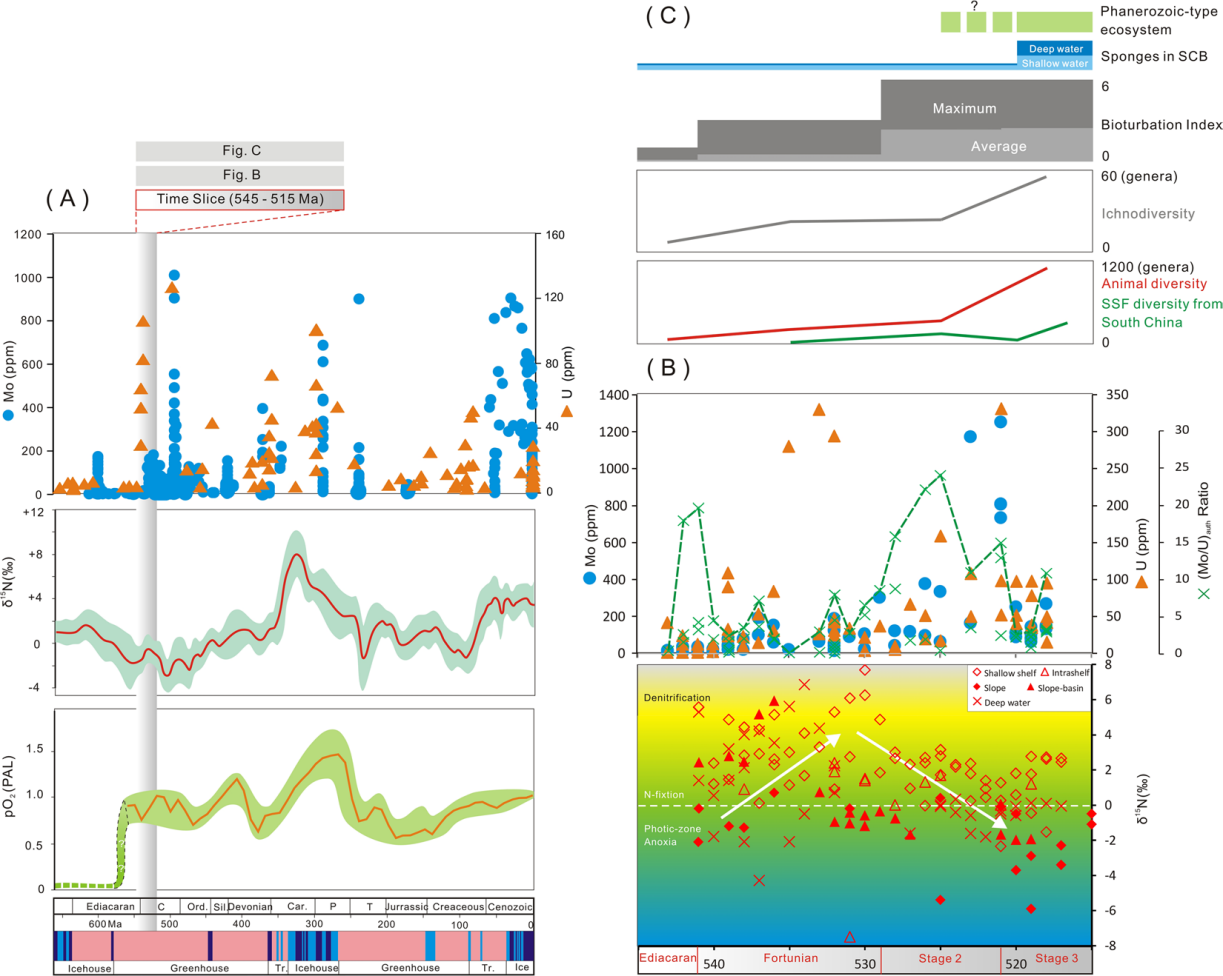
Integrated compilation of diverse geochemical indices with the biodiversity data. (**A**) Geochemical indices through geologic time. Mo concentrations were picked from refs 4 and 14, and time-averaged U concentrations were selected from the data of ref. 5, including data of this investigation. The composite curve of secular trends of δ^15^N are according to ref. 24. The curve of atmospheric oxygen levels relative to present is according to refs 2, 3 and 7. (**B**) Concentrations of Mo and U and Mo_EF_/U_EF_ through time (541–515 Ma); compilation of δ^15^N values from a range of depositional environments in the Early Cambrian Yangtze Sea (541–515 Ma). N isotopes are compiled from refs 20, 21 and this study. (**C**) Comparative bioturbation index and ichnodiversity data (referred to ref. 13); animal diversity from ref. 42 and SSF diversity data from ref. 43. The ages of glacial and green-house intervals are according to ref. 24. The white arrows in (**B**) represent the overall variation tendency of δ^15^N values.

An overview of geochemical evidences from secular and global perspectives, may tell of slightly different evolution history of atmosphere-ocean chemistry (Fig. 6). The sharp rise of oxygen levels, featured by great oxidation events (GOEs) during the Proterzoic, are also recorded by the highly increasing access of trace metals like Mo and U in the ocean^4, 5^. Recent studies^24, 25^, including results of the present investigation, have presented secular coupling records of N isotopes and inferred atmospheric oxygen levels for the Ediacaran-Cambrian boundary (Fig. 6A). Strong enrichment of Mo and U and negative N isotope trends in deposits that accumulated along the Yangtze Platform suggest that anoxic conditions existed in deep waters until Stage 3. Secular N isotope records provide evidence for progressively enhanced denitrification in the water column (highest to 8‰). Isotopic trends of diminishing values can be related to normal marine N cycling in oxygenated shallow water. Alternatively, in deep waters, reductions of δ^15^N may reflect accumulation of sediment under strongly reducing conditions, an interpretation buttressed by strong concentrations of redox-sensitive trace metals, including Mo and U (Fig. 6B). Though the existence of euxinic bottom-water conditions are challenged by published Fe speciation data, the establishment of photic zone anoxia along the slope on the eve of extensive metazoan radiation linked to oceanic oxygenation requires much more attention.

The Cambrian explosion was accompanied by increasing ichnodiversity that appears to have been unrelated to the appearance of body-fossil assemblages, including the Chengjiang Fauna^13^. Further, the establishment of suspension-feeding communities during Cambrian stage 3 is believed to have triggered the subsequent evolution of large metazoans and played a critical role in nutrient cycling. Increasing levels of bioturbation associated with the Cambrian explosion (Fig. 6C) would likely have resulted in more robust bioirregation and related oxygenation of benthic habitats. However, geochemical evidence of the present study suggest that deep waters, even on the shelf margin, remained anoxic (ferruginous) in the face of strengthening bioturbation^38^. Alternatively, the rise of body-size of animals during Early Cambrian time has been related to increasing ambient atmospheric oxygen concentrations^8^ and the consequent establishment of an enhanced early animal food web^9^. Thus, the early development of a Phanerozoic-type ecosystem should have oxygenated the ocean during the Cambrian stage 3. But oceanic oxygenation appears to have lagged in slope regions of the basin relative to the oxygenated shallow-water environments. The Cambrian explosion may have entailed not only rapid faunal diversification and ecosystem modification, but an ecological expansion from the oxygenated shelf to the anoxic deep-water realm as bottom-water oxygen levels increased. The described relationship of animals and environment comprises a complicated feedback, rather than a simple cause and effect link. Future work should focus on the association of species expansion and bioturbation with global spatial-temporal variability in ocean chemistry.

## Methods

The sampling strategy was designed to provide high resolution coverage of the Ediacaran-Cambrian boundary to Cambrian stage 3. Dozens of samples were analyzed for nitrogen isotopes, trace element concentrations and TOC (Table 1). Samples were selected from fresh exposures free of nodules. Analytical work was carried out at the Beijing Research Institute of Uranium Geology. All samples were crushed to powder (<200 mesh) before processing. TOC values were obtained using an ELTRA CS580-A carbon-sulfur analyzer and reported as weight percent.

Samples selected for N analysis were first analyzed for the total nitrogen (TN) content. Only those samples in which TN > 0.012 mg (calculated weight) were deemed capable of providing reliable nitrogen isotope results. These samples were analyzed on a Finnigan MAT-253 stable isotope mass spectrometer. Nitrogen isotope (δ^15^N) values were calculated according to the formula, δ^15^N = ((^15^N/^14^N_sample_)/(^15^N/^14^N_air_) − 1) * 1000‰.

Samples analyzed for N isotopes were also analyzed for major and trace element concentrations. Major element analysis was carried out using an Axios mAX X-ray fluorescence spectrometer with results reported as weight percent of oxides. Trace elements were measured by on ELEMENT XR ICP-MS instrument. Analytical uncertainties for analyzed elements was less than 5%. Enrichment factors (EF) were calculated as per the following; X (EF) = (X/Al)_sample_/(X/Al)_PAAS_, where X is the concentration of an element of interest and PAAS refers to post-Archean average shale compositions of Taylor and McLennan^39^. Authigenic trace element concentrations were calculated as follows: X_auth_ = X_sample_ − (X/Al)_detr_ * Al_sample_, where detrital element concentrations are those of the average upper continental crust of McLennan^40^.

## Electronic supplementary material

Supplementary Information

Dataset 1
