# Supplementary material for: Heterogenous oceanic redox conditions through the Ediacaran-Cambrian boundary limited the metazoan zonation
Source: Sci Rep. 2017 Aug 17;7:8550. doi: 10.1038/s41598-017-07904-3 (PMC5561082; doi:10.1038/s41598-017-07904-3)

Heterogenous oceanic redox conditions through the Ediacaran-Cambrian boundary limited the metazoan zonation

Junpeng Zhang [1,2,*], Tailiang Fan [3], Yuandong Zhang [1,2], Gary G. Lash [4], Yifan Li [3,] and Yue Wu [5]

[1] Key Laboratory of Economic Stratigraphy and Palaeogeography, Chinese Academy of Sciences, Nanjing 210008, China

[2] Nanjing Institute of Geology and Palaeontology, Chinese Academy of Sciences, Nanjing 210008, China

[3] School of Energy Sources, China University of Geosciences, Beijing 100083, China

[4] Department of Geology and Environmental Sciences, State University of New York, Fredonia, NY 14063, USA

[5] Sinopec Petroleum Exploration and Production Research Institute, Beijing 100083, China

1. Geological setting

1.1 Paleogeography

The South China Block (SCB) formed in the Early Neoproterozoic largely by the collision of the Yangtze Block and the Cathaysia Block[1]. Rifting of the SCB in Late Neoproterozoic time formed the Nanhua Basin, an intracontinental rift basin[2]. Paleogeographic reconstructions place the SCB close to the equator along the north margin of East Gondwana at 540 Ma though its position north or south of the equator remains open to debate[3,4]. The SCB experienced a tropical climate in Early Cambrian time as suggested by inferred seawater temperatures of ~26℃ calculated from stable oxygen isotope data collected from analysis of SSFs[5]. The Yangtze Block remained a craton from the Late Ediacaran through the Early Cambrian time, comprising the passive continental margin, transition belt and deep-water Nanhua Basin[6]. With the exception of intra-shelf depressions, also referred to as intra-shelf basins, the Yangtze Sea is believed to have been well connected with the global ocean[6,7]. Late Ediacaran transgression of the sea resulted in the widespread accumulation of marine sediments. Shelf facies deposited during the Early Cambrian comprised carbonates intercalated with fine-grained clastic layers; coeval slope and deep-water basinal facies are dominated by black shale and chert (Fig.1).

1.2 Studied sections

1.2.1 Zhongnan section

The Zhongnan section is located in Songlin town, ~ 23 km west of Zunyi city (N 27°41.344′N, 106°40.599′E), not far from the section described by previous studies[8,9]. The studied section is newly exposed by recent mining operations and is readily correlated with the earlier described section (Fig. S1A, B). The Upper Ediacaran dolomitic Dengying Formation is overlain by basal Cambrian deposits of the Niutitang Formation, which comprises a lower interval of phosphatic rock, overlain by a thin layer of pyritiferous shale that is, in turn, overlain by an approximately 20 cm-thick zircon-rich black tuff[10]. The ash layer passes upward into black siliceous mudstone containing as much as 14% total organic carbon (TOC). The carbonaceous unit hosts a V ore layer and more conspicuous Ni-Mo sulfide higher in the unit (Fig. S2). The organic-rich shale is overlain by grey to green silty shale (Fig. S2). Diverse sponges that comprise the Niutitang Sponge Fauna are present in the black shale above the sulfide layer, and trilobites are common to the upper part of the silty shale. A supposed unconformity between the Niutitang and Dengying Formation reflects a hiatus of at least 3 Ma according to the SHRIMP U-Pb age of the tuff layer and the equivalent strata from other sections[9,11]. Thus the age of basal strata in this section is preliminarily set as

538 Ma, yielding a long hiatus. The Zhongnan section is interpreted to have accumulated in an intra-shelf basin[6].

1.2.2 Nangao section

The Nangao section is situated in Nangao town, ~ 25 km northeast of Danzhai city (26°22.895′N 107°52.86′E). Presence of Ni-Mo sulfide and V ore layers in the Nangao section permits its correlation with other investigated sections (Fig. S2). The initial Cambrian deposits that accumulated over the dolomitic Dengying Formation comprise a thin succession of interbedded chert and shale known as the Laobao Formation, which is overlain by a thin interval of phosphatic rock at the bottom of the Niutitang Formation (Fig. S1C)[12]. However, the bulk of the Niutitang Formation is organic-rich mudstone containing sponge spicules (Fig. S2). Overlying organic-lean silty shale contains abundant sponge fossils typical of the deep-water Niutitang Sponge Fauna. A massive dolomitic turbidite in the Niutitang Formation (Fig. 2) may reflect sediment transport from an adjacent carbonate margin suggesting accumulation in a slope environment.

1.2.3 Longbizui section

This Longbizui section is located in Morong town, ~ 18 km southwest of Guzhang (28°29.929′N, 109°50.533′E), which is the location of the GSSP of the Guzhangian stage (Fig.S1F). The Liuchapo Formation comprises thick-bedded chert intercalated with thin layers of shale at its top (Fig. S2). The Precambrian-Cambrian boundary in the Longbizui section is assigned to the upper part of the Liuchapo Formation (Fig. S2), according to recent fossil evidence[13]. The overlying Niutitang Formation is recognized by the presence of phosphatic shale overlain by phosphate nodule-bearing, organic-rich black shale that passes up-section into interbeded chert and shale (Fig. S2). The Ni-Mo sulfide ore layer was recognized immediately below the contact of black shale and overlying chert (Fig. S2). Sparse deep-water sponge fossils have been recovered from the upper part of Niutitang Formation. The Longbizui section is interpreted to be a slope-basin succession that likely accumulated in water deeper than that in which sediments of the Nangao section were deposited.

1.3 Regional stratigraphic correlations

The generally thin basal Cambrian succession that accumulated in slope and basinal environments preserves the history of a critical geological interval (Meishucunian-early Qiongzhusian, or Fortunian-stage 3) of Earth history. The Ediacaran-Cambrian boundary is traditionally placed at the transition from widespread dolostone of Dengying Formation to overlying deposits comprised of variable amounts of siliceous dolostone, shale, and chert interpreted to be shallow carbonate shelf to deep-water basinal sediments (Fig. S2). The boundary appears to be roughly coincident with a pronounced negative carbon isotope excursion, the Basal Cambrian Carbon-isotope excursion or BACE[14]. In deep-water facies, however, the negative excursion is found in a shale-chert horizon beneath the phosphate nodule-bearing shale of Niutitang Formation thereby placing the basal boundary of

the Cambrian in the upper Liuchapo Formation (Fig. S2). Thus, the Liuchapo Formation is not considered to be the equivalent of the Dengying Formation.

The age of the Ediacaran-Cambrian boundary is constrained at 541 Ma[15]. Recent geochronological data have helped to calibrate the Lower Cambrian stratigraphy of the studied sections. The tuff layer immediately below the phosphatic layer of the Zhongnan section has yielded U-Pb age of 532.3 ±0.7 Ma[9] (Fig. S2). The sulfide layer of the same section yields an age of 521.5 ±5 Ma by Re-Os geochronology[16] (Fig. S2). Further, the slope-basin sections equivalent to Nangao and Longbizui sections have yielded a set of U-Pb zircon ages of 524.2 ±5.3 Ma and 522.3 ±3.7 Ma for the tuffaceous bed below the sulfide ore layer, and 542.6 ±3.7 Ma for the tuff deposit in the upper part of Liuchapo Formation of the latter section[17] (Fig. S2). The generally consistent approximately 521 Ma age of the widespread Ni-Mo sulfide layer reveals its potential as a robust stratigraphic and geochronologic marker. Indeed, the Ni-Mo layer facilitates the correlation of slope and basinal facies with thick, fossil-rich strata of the carbonate platform (Fig. S2).

Shallow platform deposits can be readily correlated by use of widespread, well-preserved SSFs. SSF assemblages identified in carbonate rocks intercalated with chert and shale of the Three Georges Area section permit the accurate correlation of these deep-shelf deposits with shallow-shelf deposits exposed in the Xiaotan section[18,19] (Fig. S2). Although sponge fossils are common to slope and deep-water successions (i.e., Niutitang Sponge Fauna), sparse sponge spicules have been recovered (Fig. S2). Still, the diverse sponge fossils are common to the grey and black shale of the upper Niutitang Formation providing an additional means of stratigraphic correlation. Thus, this critical period can be divided into three intervals according to lithological characteristic along with variations in biostratigraphy and chemostratigraphy (Fig. S2). The first interval are featured by the onset of BACE and widespread deposition of chert. While the interval 2 gets clearly identified with the occurrence of Ni-Mo sulfide layer in the slope and basin and ZHUCE recovered from carbonates in the shallow shelf. Herein the interval 3 begins above the sulfide layer, with widespread shale containing a variety of sponges in both slope and deep-water basin. This dividing would provide an episodic view of the ocean chemistry combined with the interpretation of geochemical evidences in the text.

2. References

1. Shu, L.S., Faure, M., Yu, J.H., Jahn, B.M. Geochronological and geochemical features of the Cathaysia block (South China): New evidence for the Neoproterozoic breakup of Rodinia. *Precambr. Res.* 187, 263-276 (2011).

2. Li, X.H., *et al.* Amalgamation between the Yangtze and Cathaysia Blocks in South China: Constraints from SHRIMP U–Pb zircon ages, geochemistry and Nd–Hf isotopes of the Shuangxiwu volcanic rocks. *Precambr. Res.* 174, 117–128 (2009).

3. Scotese, C.R., 2004. Paleomap Project. http://www.scotese.com.

4. Li, Z.-X., Evans, D.A.D., Halverson, G.P. Neoproterozoic glaciations in a revised global palaeogeography from the breakup of Rodinia to the assembly of Gondwanaland. *Sedi. Geol.* 294, 219-232 (2013).

5. Chen, Y., Jiang, S., Ling, H., Yang, J., Wan, D. Isotopic compositions of small shelly fossil Anabarites from Lower Cambrian in Yangtze Platform of South China: Implications for palaeocean temperature. *Progress in Natural Science* 17, 1185-1191 (2007).

6. Zhang, J., Fan, T., Algeo, T.J., Li, Y., Zhang, J. Paleo-marine environments of the Early Cambrian Yangtze Platform. *Palaeogeogr. Palaeoclimatol. Palaeoecol.* 443, 66-79 (2016).

7. Cremonese, L., Shields-Zhou, G.A., Struck, U., Ling, H.-F., Och, L.M. Nitrogen and organic carbon isotope stratigraphy of the Yangtze Platform during the Ediacaran–Cambrian transition in South China. *Palaeogeogr. Palaeoclimatol. Palaeoecol.* 398, 165-186 (2014).

8. Wille, M., Nägler, T.F., Lehmann, B., Schröder, S., Kramers, J.D. Hydrogen sulphide release to surface waters at the Precambrian/Cambrian boundary. *Nature* 453, 767-769 (2008).

9. Jiang, S.Y., et al. Early Cambrian ocean anoxia in South China. *Nature* 459, E5-6; discussion E6 (2009).

10. Pi, D.-H., Liu, C.-Q., Shields-Zhou, G.A., Jiang, S.-Y. Trace and rare earth element geochemistry of black shale and kerogen in the early Cambrian Niutitang Formation in Guizhou province, South China: Constraints for redox environments and origin of metal enrichments. *Precambr. Res.* 225, 218-229 (2013).

11. Compston, W., Zhang, Z., Cooper, J.A., Ma, G., Jenkins, R.J.F. Further SHRIMP geochronology on the early Cambrian of South China. *Am. J. Sci.* 308, 399-420 (2008).

12. Yang, Aihua, Zhao, Yuanlong; Zhu, Maoyan; Cui, Tao; Yang, Kaidi. SPONGES FROM THE EARLY CAMBRIAN NIUTITANG FORMATION AT DANZHAI, GUIZHOU AND THEIR ENVIRONMENTAL BACKGROUND. *Acta Palaeo. Sin.* 49, 348-359 (2010).

13. Wang Yue, H.Z., Chen Hongde, Hou Mingcai, Yang Yanfei, Du Bingying. Stratigraphical Correlation of the Liuchapo Formation with the Dengying Formation in South China. *Journal of Jilin University (Earth Science Edition)* 42, 328-334 (2012).

14. Zhu, M.-Y., Babcock, L.E., Peng, S.-C. Advances in Cambrian stratigraphy and paleontology: Integrating correlation techniques, paleobiology, taphonomy and paleoenvironmental reconstruction. *Palaeoworld* 15, 217-222 (2006).

15. Gradstein, F.M., Ogg, J.G., Schmitz, M.D., and Ogg, G.M. The Geological Time Scale 2012, Elsevier, 2 vols., 1144 p (Amsterdam, 2012).

16. Xu, L., Lehmann, B., Mao, J., Qu, W., Du, A. Re-Os Age of Polymetallic Ni-Mo-PGE-Au Mineralization in Early Cambrian Black Shales of South China--A Reassessment. *Econo. Geol.* 106, 511-522 (2011).

17. Chen, D., Zhou, X., Fu, Y., Wang, J., Yan, D. New U-Pb zircon ages of the Ediacaran-Cambrian boundary strata in South China. *Terra Nova* 27, 62-68 (2015).

18. Cremonese, L., et al. Marine biogeochemical cycling during the early Cambrian constrained by a nitrogen and organic carbon isotope study of the Xiaotan section, South China. *Precambr. Res.* 225, 148-165 (2013).

19. Guo, J., Li, Y., Li, G. Small shelly fossils from the early Cambrian Yanjiahe Formation, Yichang, Hubei, China. *Gondwana Res.* 25, 999-1007 (2014).

20. Yang, X., Zhu, M., Guo, Q., Zhao, Y. Organic Carbon Isotopic Evolution during the Ediacaran-Cambrian Transition Interval in Eastern Guizhou, South China: Paleoenvironmental and Stratigraphic Implications. *Acta Geol. Sin.* 81, 194-203 (2007).

3. Figures with captions

Fig. S1 Pictures to show formations and their boundaries in different sections in the Yangtze Platform. (A) fresh outcrop in Zhongnan equivalent to the old one; (B) the old Zhongnan section presented in many previous studies; (C) and (D) distinct formation contacts in the Nangao section; (E) bedded chert and dolostone overlying thick dolostone of the Dengying Formation, Three Gorges area; (F) slope facies dominated by chert and shale, Longbizui section. Fm.: Formation. All the photographs were taken by the author and co-authors.

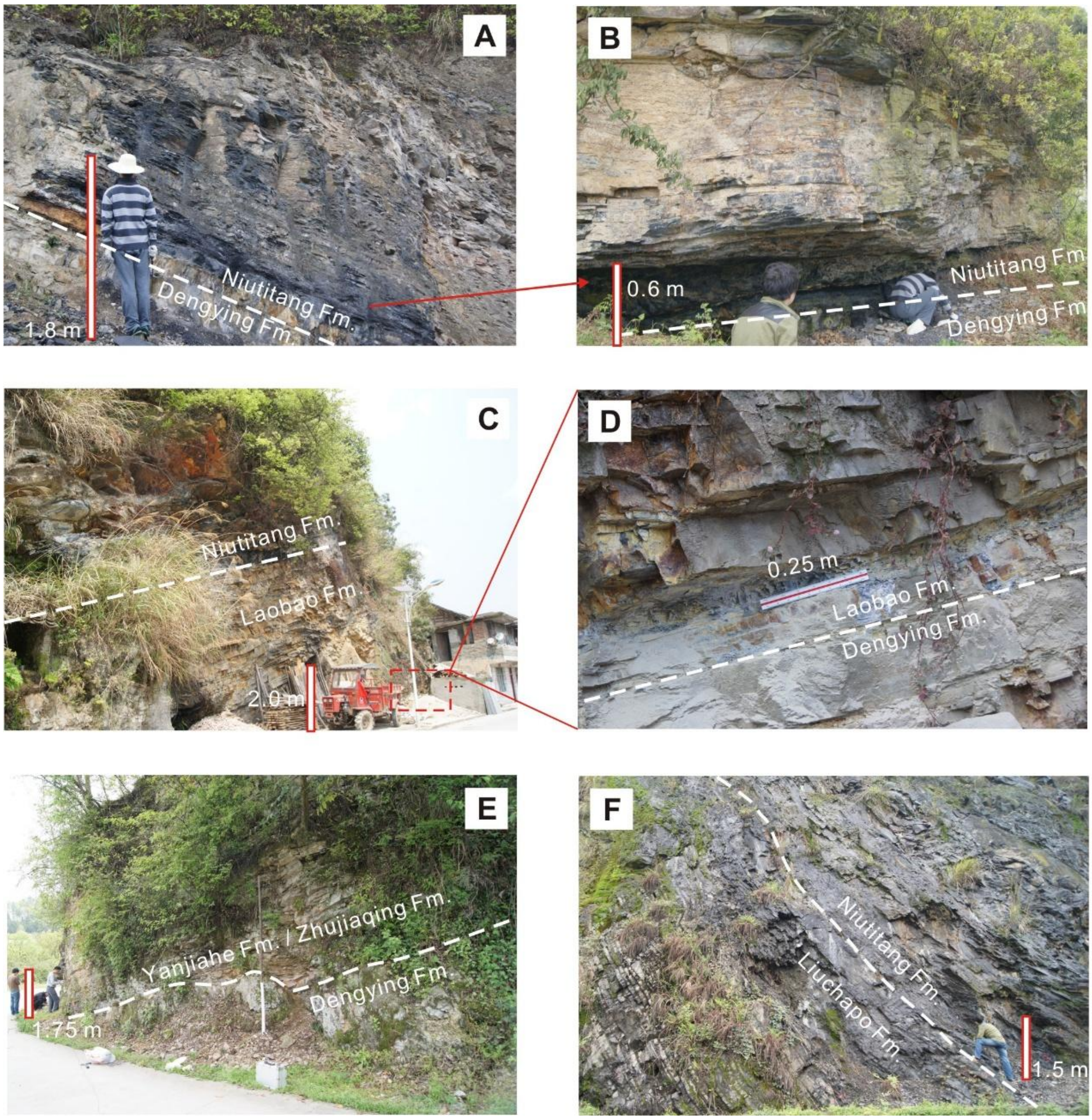

Fig. S2 Regional stratigraphic correlations, with published chemostratigraphy and biostratigraphy data. Organic carbon isotope data are compiled from Ref.20 and 7; records of small shelly fossil assemblage are from Ref.18 and 19; occurrences of sponge fossils are from Ref.12; chronological data are shown with their sources. BACE: Basal Cambrian Carbon isotope Excursion[14].

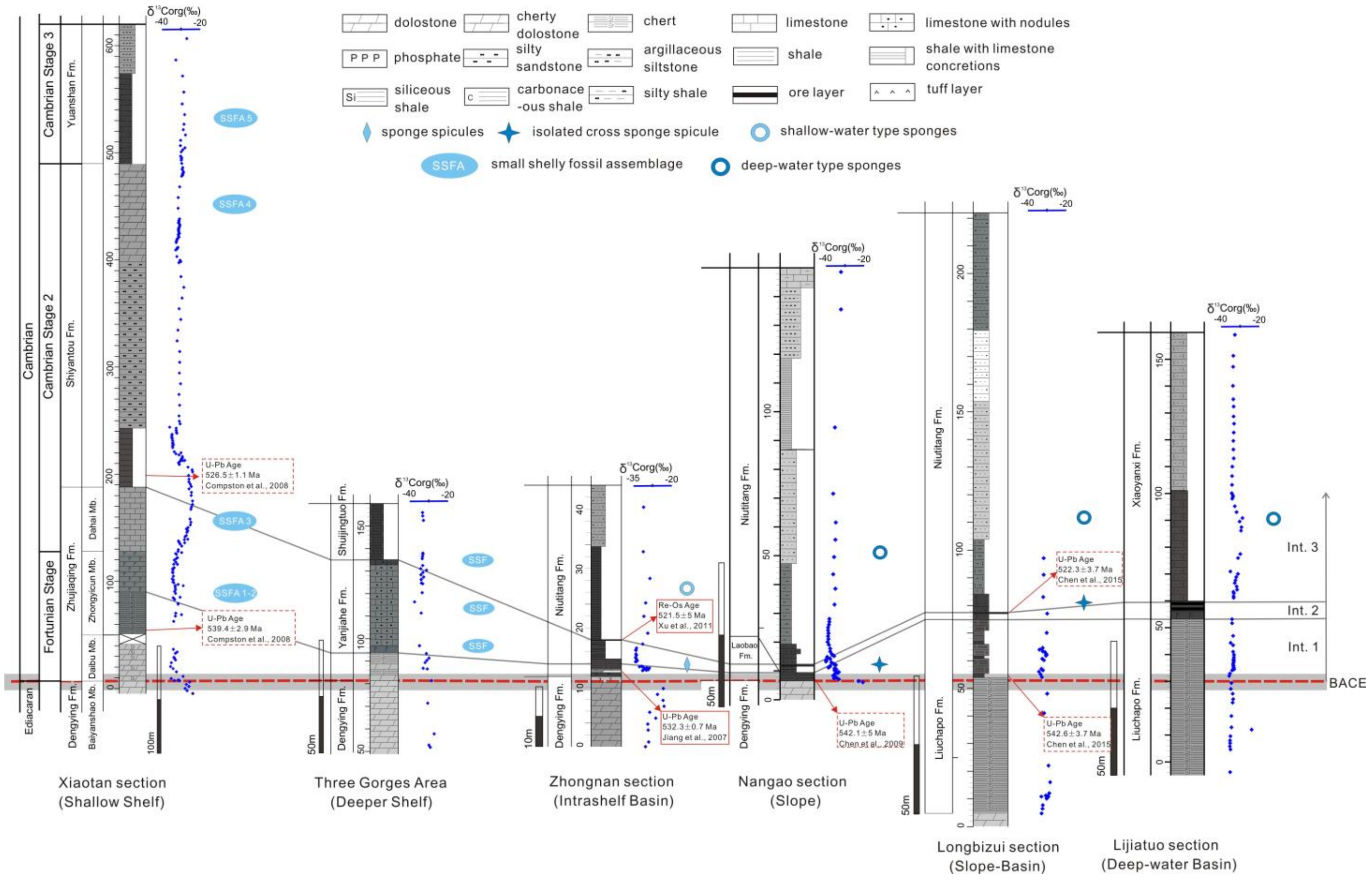

Supplement: Supplementary file 1 — Supplementary Information [file 41598_2017_7904_MOESM1_ESM.pdf]
